# Impact of cultivation strategy, freeze-drying process, and storage conditions on survival, membrane integrity, and inactivation kinetics of *Bifidobacterium longum*

**DOI:** 10.1007/s12223-020-00815-3

**Published:** 2020-08-27

**Authors:** Regina Haindl, Alexandra Neumayr, Anika Frey, Ulrich Kulozik

**Affiliations:** Chair of Food and Bioprocess Engineering, Technical University of Munich, Weihenstephaner Berg 1, Freising-Weihenstephan, Germany

**Keywords:** *Bifidobacterium longum* ssp. *longum* Reuter 1963, Maltodextrin, Membrane preservation, Probiotics, Protectants, Storage temperature

## Abstract

*Bifidobacterium longum*, one of the main microorganisms in the human gut, is used as an adjunct to lactic acid starter cultures or sold as a probiotic product. Therefore, *Bifidobacterium longum* cell suspensions get freeze-dried with protective additives to prevent activity losses. To date, investigations covering growth and inactivation kinetics of *Bifidobacterium longum* during the whole process (cultivation, drying, and storage) have been lacking. In this study, the effect of cultivation conditions and shelf temperature as well as the influence of protectants (maltodextrin, glucitol, trehalose) at various concentrations on cell survival during freeze-drying was assessed. Drying was followed by a storage at + 4 °C and + 20 °C for 70 days to evaluate inactivation kinetics. The impact of the different factors was assessed by measuring surival rate and residual moisture content at various points of time over the whole process. In parallel cell membrane integrity and glass transition were determined to reveal inactivation effects. Cultivation strategy had a strong influence on survival with a huge potential for process improvement. A pH of 6.0 at the growth optimum of the strain provides better conditions regarding cell survival after drying than free acidification (non-regulated pH conditions). During the drying step, membrane leakage due to the removal of water is the main reason for the inactivation in this process step. In this study, the highest survival of 49% was obtained with cells dried at + 35 °C shelf temperature with an addition of maltodextrin (75% bacterial dry matter, w/w). The results show that *Bifidobacterium longum* cells are mostly inactivated during drying, whereas storage conditions at + 4 °C with an addition of 75% BDM maltodextrin relative to bacterial dry mass prevent cell loss completely.

## Introduction

Bifidobacteria, attributed to the phylum Actinobacteria, are one of the core microorganisms in the human intestinal microbiome. Numerous studies have demonstrated their probiotic activities, their impact on the microbiome, and their effect on human health (Quigley 2017). Bifidobacteria produce organic substances such as lactic and acetic acids. These acids lower the pH in the colon and promote the growth of other beneficial intestinal bacteria (Sugahara et al. 2015), as well as prevent the colonization of the gut by pathogenic microorganisms (Gibson and Wang 1994). Bifidobacteria can further improve lactose utilization in the case of malabsorption by lactose-intolerant individuals (Kailasapathy and Chin 2000). Typical species in the large intestine include *Bifidobacterium adolescentis*, *Bifidobacterium breve*, and *Bifidobacterium longum*. The latter is abundant in the human gut and found at a concentration of 10^8^ CFU/g (Matsuki et al. 2004).

In industry, *Bifidobacterium longum* is used as an adjunct culture in fermented dairy products. It is also distributed as a liquid or dried probiotic nutritive supplement. Such nutritive supplement cultures are produced as starter cultures in large-scale cultivation systems. In order to be able to store and flexibly use these cultures, the obtained cells are freeze-dried. The technical production process of microorganisms, therefore, includes cultivation, drying, and storage. Each step has different effects on the final number of active cells in the finished product. To prevent major losses of active cells and to minimize inactivation, processing conditions along the entire chain of production must be carefully chosen. Up until now research often concentrates on one single step of the production process. To work efficiently, it is necessary to regard the entire chain to develop a process obtaining a high cell count and survival at the end. Various works report on the effect of cultivation conditions on bifidobacteria survival during the drying step. A higher survival rate was achieved when the pH during cultivation was held at the optimal value of 6.0 for *Bifidobacterium lactis* (Bauer et al. 2012; Kiviharju et al. 2005). Also, recommendations so far suggest that cell harvesting should be carried out during the late-logarithmic or early stationary phase as the cells here are the most resistant to drying (Saarela et al. 2005). However, methods to produce and preserve microorganisms differ. As a result, survival rates reported in previous literature are not readily comparable. The commonly used method during industrial probiotic production of cells is freeze-drying (Champagne et al. 1991; Saarela et al. 2005; Saarela et al. 2006; Shamekhi et al. 2013; Wang et al. 2004). During drying, the water surrounding the cell as well inside the bacterial cell membrane is removed. Consequently, membrane leakage occurs and the bacterial cell is not able to recover and restart growth. This is the main reason for cells getting inactivated. During drying and storage, protectants can be added to prevent inactivation. Protectants like maltodextrin, glucitol, or trehalose have already been used for different lactobacilli strains (Ambros et al. 2018a; Oldenhof et al. 2005; Sohail et al. 2013). These molecules can act as cryoprotectants (Dianawati et al. 2013) or through different protection mechanisms as water replacement or the glassy state (Santivarangkna et al. 2008). Studies on bifidobacteria strains have demonstrated that both the choice of protective agent and the processing conditions influence cell survival in a strain-dependent manner (Yeung et al. 2016). Consequently, the individual strains’ properties must be considered when selecting the conditions for the drying process.

During storage, among others, the environmental temperature on freeze-dried *Bifidobacterium longum* has an influence on the survival rate as examined by several researchers (Bruno and Shah 2003; Reilly and Gilliland 1999). Furthermore, a low storage temperature seemed to be appropriate for microencapsulated cells of *Bifidobacterium longum* (Hsiao et al. 2004). At storage, low relative humidity is required to obtain high survival rates (Abe et al. 2009; Min et al. 2017).

However, the behavior during storage of non-encapsulated cells in combination with the addition of protectants, as well as the influence across all process unit operations including cultivation, drying, storage, and the overall rate of inactivation, has not yet been studied further. An investigation of how these factors in combination affect the survival of *Bifidobacterium longum* is still missing. Therefore, the purpose of this study is to investigate key factors in survival and to characterize and to improve a *Bifidobacterium longum* Reuter 1963 production process with regard to raise the level of survival. We set out to combine the factors involved in inactivation and determine how cell count evolves over the whole process including storage of the freeze-dried culture. Furthermore, the interaction among protectant and temperature during storage was investigated. A storage time of 70 days was chosen, because this was considered long enough to identify storage-related effects on inactivation. Our hypothesis therefore is that by assessing the impact of factors, the rate of survival could be raised and these effects can be better understood by measuring glass transition and cell membrane integrity in parallel.

## Materials and methods

### Bacterial culture and fermentation process

*Bifidobacterium longum* ssp. *longum* Reuter 1963 from the DSMZ (German Collection of Microorganisms and Cell Cultures, Braunschweig, Germany), hereafter referred to as *B. longum* Reuter 1963, was chosen as the test strain.

One batch of *B. longum* Reuter 1963 was used as the inoculum for all experiments. A bioreactor BioStat C (Sartorius Stedim Biotech GmbH, Göttingen, Germany) containing 8 L of broth was inoculated to obtain a starting cell concentration of *A*_600_ = 0.3 (3 · 10^8^ CFU/mL). The cells were grown in MRSc medium (MRS medium with 1 g/L cystein) at + 37 °C, with a mixing speed of 80 rpm and sparged with 0.1 L/min N_2_. The initial pH of the medium was adapted to pH 6.0. In the cultivation process, the pH was either not regulated (free acidification) or adjusted by adding 2 mol/L NaOH or 0.5 mol/L HCl to maintain pH 6.0. These conditions were found to be of major importance by previous works (Bauer et al. 2012).

### Sample preparation and protectants

**Fig. 1:**
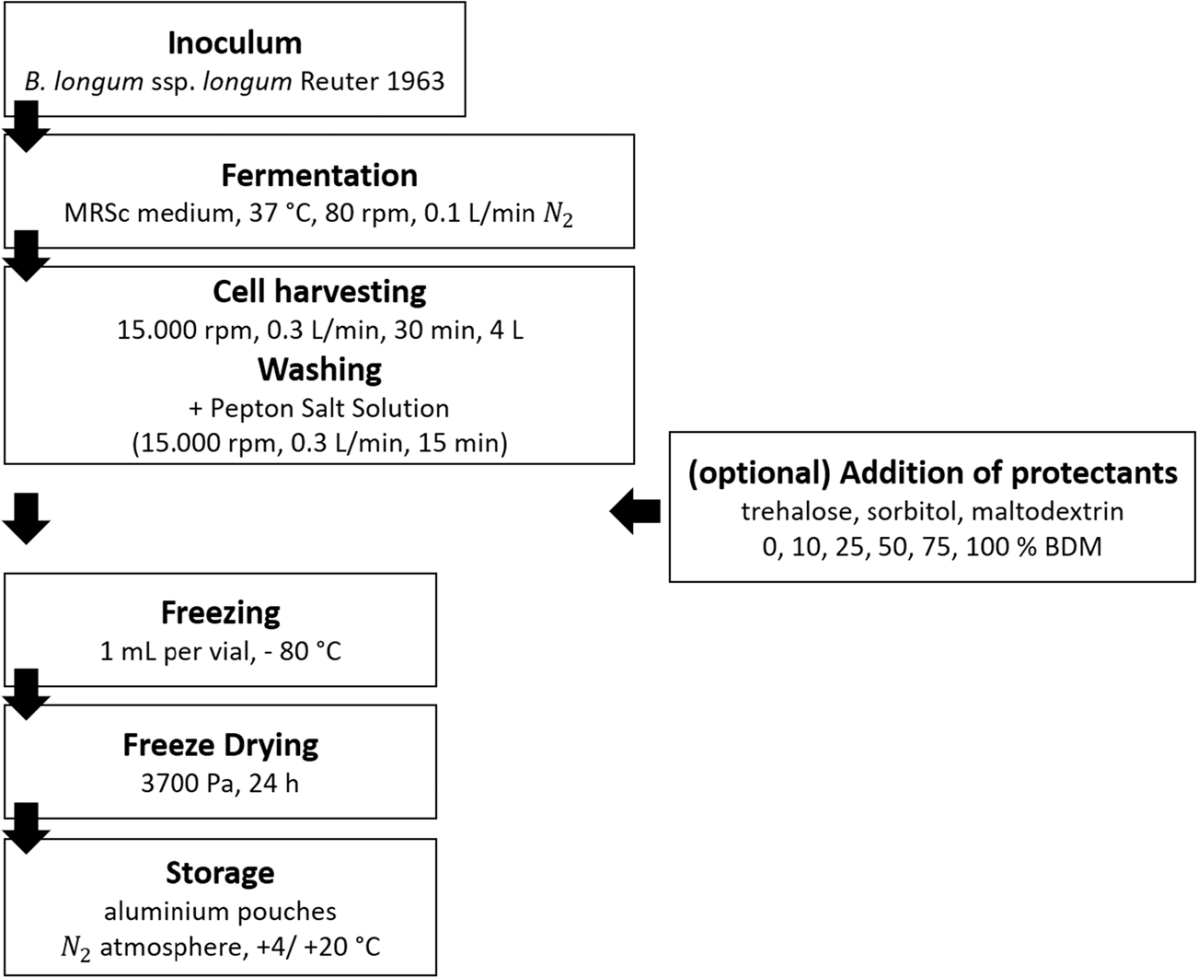
Process scheme applied in this study

Following cultivation, the cell suspension was centrifuged using a Heraeus™ Biofuge™ Stratos™ Centrifuge (Thermo Fisher Scientific, Waltham, USA; 15,000 rpm, 0.3 L/min, 30 min) to enable cell harvesting. Then, the cells were washed by adding peptone salt solution (1 g/L peptone from casein; 8.5 g/L NaCl; 0.3 g/L KH_2_PO_4_; 0.6 g/L Na_2_HPO_4_·2H_2_O) and centrifuged again under the same conditions for another 15 min. After homogenizing the cell pellet, the protectants maltodextrin (dextrose equivalent 4.0–7.0; Sigma-Aldrich, Steinheim, Germany), D(+) trehalose (Sigma-Aldrich, Steinheim, Germany), and glucitol (D(-) sorbitol, Merck KGaA, Darmstadt, Germany) were added in several concentrations (0, 10, 25, 50, 75, and 100%) related to the bacterial dry matter (BDM, w/w). Afterwards, 1-mL aliquots of the suspensions were pipetted into freeze-drying glass vials and frozen in a − 80 °C freezer BF-U538 (Buchner Labortechnik, Pfaffenhofen an der Ilm, Germany). The process scheme is illustrated in Fig. 1.

### Freeze-drying

Freeze-drying was conducted in three successive steps using a Delta 1-24 LSC pilot plant dryer (Christ GmbH, Osterode, Germany). The chamber pressure was kept at a constant 3700 Pa, while the shelf temperature rose in a stepwise manner (Table 1).

**Table 1:** Drying protocol for preservation of *B. longum* Reuter 1963 with the temperature in process step 3 adjusted to + 25, + 35, or + 40 °C

Process step	Chamber pressure (Pa)	Shelf temperature (°C)	Duration (h)
1	3700	− 10	12
2	3700	+ 10	6
3	3700	+ 25/+ 35/+ 40	6

The temperatures of the shelf and inside the product were controlled with internal sensors to avoid exceeding the set temperature limits. During freeze-drying at a constant chamber pressure, the amount of heat brought into the product is the driving force. The heat transfer rate *dQ*/*dt* is dependent on several factors as shown in Eq. 1:

1 $$ \frac{dQ}{dt}={A}_{\mathrm{v}}\cdotp {K}_{\mathrm{v}}\cdotp \Delta  \vartheta $$

where *A*_v_ is the contact surface of the used vial and *K*_v_, the heat transfer coefficient of the glass vial are geometry and material dependent and constant, respectively. Consequently, the amount of heat *Q* that is transferred to the product in a certain time intervall is only dependent on Δ*ϑ*, the difference of temperature between the product and the shelf. The product temperature cannot be regulated separately, but is dependent on the shelf temperature, chamber pressure, and consequently moisture and heat flow rate. The shelf temperature was adjusted stepwise to remove surface and structural water molecules. Removing structural water required additional energy and, therefore, the shelf temperature during the third step of the process was adjusted to + 25, + 35 or + 40 °C. Details regarding the choice of these conditions will be discussed later.

### Packaging and storage conditions

After drying, approximately 0.5 g per sample was packed in light-proof aluminum bags (PET/ALU/PE—12/12/75 μm; oxygen permeability < 0.0001 cm^3^/m^2^ day hPa) under nitrogen atmosphere (< 5 Vol.-ppm H_2_O). They were stored at + 4 °C or + 20 °C for 7, 14, 21, 28, 42, 56, and 70 days until analysis. At each time point, at least two bags were chosen for analysis.

### Analysis of residual moisture content and water activity

Both after drying and at each point of time, residual moisture content was determined. Water activity was analyzed with an A_w_ Sprint TH-500 (Novasina, Lachen, Switzerland). The residual moisture content was measured using a CEM Smart Turbo™ 5 (CEM Corporation, Kamp-Lintfort, Germany) at a maximum sample temperature of + 80 °C and 45% power input. The results obtained with the CEM device were double-checked regularly by Karl Fischer titration using TitroLine KF (Mettler-Toledo GmbH, Schwerzenbach, Germany).

### Analysis of survival and inactivation rate constant

The survival rate was determined by rehydrating the dried cell powder with sterile bi-distilled water to its initial biological dry matter (after cell harvest and concentration, before drying). The obtained suspension was then diluted with 0.25 strength Ringer’s solution and plated on MRSc agar plates (MRSc broth with 15 g/L agar-agar). These plates were incubated anaerobically for 48 h at + 37 °C. Plates with 30 to 300 colonies were included in counting colonies. The number of colony-forming units *N* per milliliter of sample (CFU/mL) was calculated according to Eq. 2.

2 $$ N=\frac{c}{n_1+\left(\ 0.1\cdot {n}_2\right)} $$

where *c* is the sum of colonies of the subsequent dilutions with *n*_1_ the number of colonies in the less diluted solution and *n*_2_ the number of colonies in the more diluted solution.

The survival rate (*SR*, %) was then calculated by the ratio of CFU as shown in Eq. 3. Hereby, *N*_0,*i*_ refers to the individual plate count before each processing step (CFU before drying or storage) and *N*_*t*_ refers to the plate count as a function of processing time and processing step.

3 $$ SR=\frac{N_t}{N_{0,i}}\cdotp 100 $$

The survival rate was determined at different points of time during the drying process and storage.

The inactivation rate during storage represents the rate of active of cells loss in a specific period of time (per day). It was determined as the logarithmic value of the ratio of the cell count at the beginning of the storage period (after drying, *N*_0_) and after particular process time points (*N*_*t*_). The residual cell count (*N*_*t*_/*N*_0_) was plotted logarithmically over time (*t*, days) for each storage temperature and protectant addition. The inactivation rate constant *k* (per day) was calculated from the slope of the linear regression of the obtained data, as shown in Eq. 4:

4 $$ \log\ \frac{N_t}{N_0}=-\frac{k\cdot t}{-2.303} $$

### Assessment of membrane preservation

To analyze cell membrane preservation, the cells were stained with fluorochromes. The fluorochrome propidium iodide (PI) is able to pass through the damaged membrane of dead cells and can thus interact with DNA within the cell. The resulting fluorescence was detected at a filter wave length of 670 nm. On the other hand, the dye thiazole orange (TO), which is able to diffuse through the intact cell membrane and interacting with both DNA and RNA, induces fluorescence of intact cells. The absorbance of TO was measured at an excitation wave length of 530 nm. Cells with a damaged cell membrane were stained by both fluorochromes.

PI (Sigma-Aldrich Chemie, Darmstadt, Germany) was dissolved to 21 μmol/L; TO (Sigma-Aldrich Chemie, Darmstadt, Germany) was used as 1 μmol/L solution. One hundred microliters of cell suspension (10^−3^ dilution) was mixed with 290 μL PBS buffer (0.2 g/L KCl; 8.0 g/L NaCl; 0.27 g/L KH_2_PO_4_; 1.42 g/L Na_2_HPO_4_·2H_2_O), 100 μL PI, and 10 μL TO solutions, homogenized, and incubated for 10 min at room temperature. After incubation, the samples were measured in a BD Accuri™ C6 flow cytometer (Accuri Cytometers, Ann Arbor, USA).

Membrane integrity (*MI*, %) was calculated as the ratio of the three differentiable groups in Eq. 5:

5 $$ MI=\frac{N_{\mathrm{intact}}}{N_{\mathrm{intact}}+{N}_{\mathrm{damaged}}+{N}_{\mathrm{dead}}}\cdotp 100 $$

Membrane preservation (*MP*, %) was calculated as the ratio of *MI* before drying (*MI*_0_) and after different time points in the process (*MI*_*t*_) as Eq. 6 shows:

6 $$ MP=\frac{MI_t}{MI_0} $$

### Analysis of glass transition temperature

The glass transition temperature of the samples after drying was measured with modulated differential scanning calorimetry Q1000 (TA Instruments, New Castle, USA). Two hermetically closed aluminum pans were used for each measurement. An empty pan was used as reference, whereas the second pan contained 10–15 μg of the dried sample. Before measurements, the system was calibrated with indium and the measuring chamber was flooded with liquid nitrogen. During the measurement, the heating rate was set to 2 K/min over a temperature range from − 60 to + 150 °C with a modulation time of 60 s and a temperature amplitude of ± 1 °C. Analysis was performed with the software TA Universal Analysis 2000 (TA Instruments, New Castle, DE, USA). Therefore, the reversing heat flow (W/g), heat flow (W/g), and nonreversing heat flow (W/g) were plotted over temperature. Onset and offset glass transition temperatures were marked by applying tangents to the break points of the plotted graph of the reversing heat flow. The inflection point was calculated and mid glass transition temperature was considered for further evaluation.

### Statistical analysis

All analyses were repeated at least in triplicate. The mean values are shown as the arithmetic mean $\overline{x}$ of the number n of all samples *x*_*i*_. The distribution of the values was calculated from the standard deviation *s* due to the random error. All graphs in the following show arithmetic means ± standard deviations. The results were tested for significance using a one-way ANOVA (*p* ≤ 0.05), followed by a Tukey post hoc analysis to reveal significant influencing parameters. The tests were conducted with the software OriginPro 2019 (OriginLab Corporation, Northampton, USA).

## Results and discussion

The hypothesis of this work is that an investigation of several relevant process settings and inactivation during all steps allows an optimization of survival. In parallel, measurements of glass transition temperature and membrane integrity will help to explain effects causing losses in viability. The most harmful steps and conditions for *B. longum* Reuter 1963 during the production process and storage will get identified. Furthermore, the interaction among protectant and termperature during storage will be investigated. High survival rates, low residual moisture contents, and a long shelf life are selected as desired variables.

### Effect of cultivation conditions on cell growth and survival after drying

**Fig. 2:**
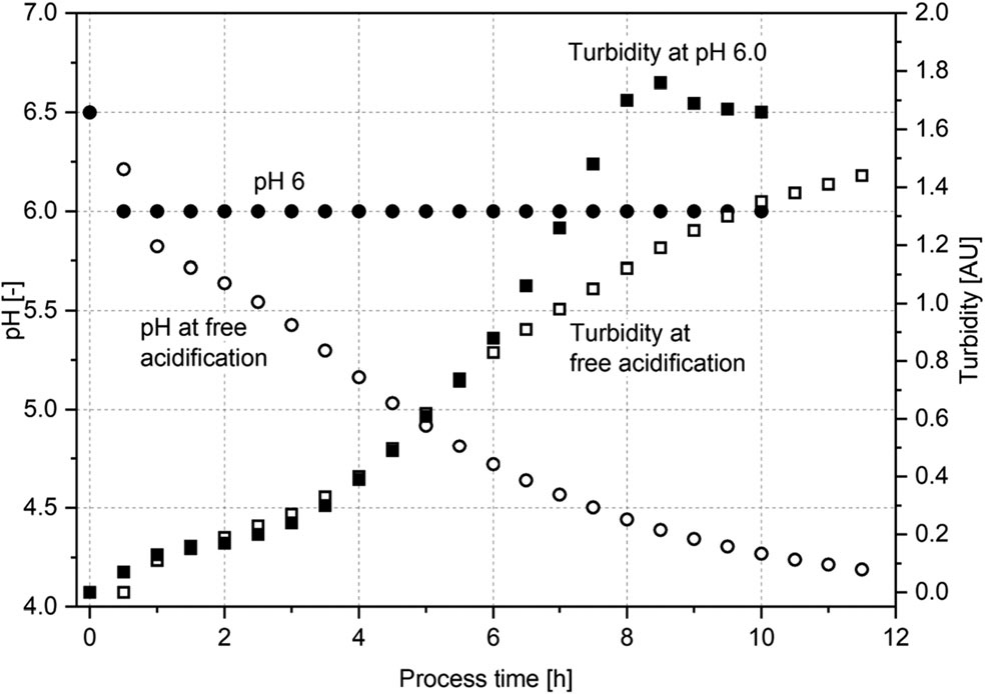
Exemplary turbidity and pH value at pH-controlled (pH 6.0) and non-regulated (free acidification) cultivation

The production process of probiotics starts with the cultivation step. In this study, the influence of the cultivation pH on cell count and survival rate after a freeze-drying process was examined. Figure 2 shows the absorbance and pH value obtained at two fermentation modes of *B. longum* Reuter 1963 cell suspensions: free acidification (non-regulated pH) and pH 6.0 (pH for optimal growth (Kiviharju et al. 2005)). Each cultivation was stopped in the early stationary phase. Harvesting cells in the early stationary phase achieves the best survival rate after drying as shown by Corcoran et al. (2004). The harvesting point was reached after 10 h at pH 6.0 and 11.5 h under non-regulated pH conditions. This is similar to times reported by Lian (2002), who also detected the stationary phase after 12–15 h when cultivating *B. longum* Reuter 1963 cells with MRS medium supplemented with 0.05% cysteine under non-regulated pH conditions. A non-regulated pH results in free acidification, induced by the production of short-chain fatty acids such as acetic or lactic acid. In the beginning, the turbidity measurements under both cultivation models were equal. After 5.5 h, however, the culture grown under free acidification conditions began to show a smaller increase in turbidity as compared with the culture kept at pH 6.0. At this point, the pH dropped below 4.8, which seems to be a critical pH for the strain since growth was restricted from that point on. This resulted in a lower turbidity value than that of the culture held at pH 6.0. After 10 h, the broth cultivated at pH 6.0 reached a turbidity of 1.66 AU. The cells cultivated without pH regulation resulted in 1.44 AU after 11.5 h. Following cell harvest by centrifugation, a comparable number of cells was established in both cases: 2 · 10^10^ CFU/mL for non-regulated cultivation and a cell count of 5 · 10^10^ CFU/mL after cultivation at pH 6.0. Then, the cells were freeze-dried (3700 Pa, 24 h, − 10/+ 10/+ 35 °C, no protectant). The resulting survival rates are shown in Table 2. A high number of cells cultivated at the optimum pH of 6.0 survived the drying process (40%). In comparison, cells cultivated under free acidification conditions were less likely to survive (0.5%). Hence, the pH control during cultivation has a significant statistical influence based on a *p* value of 0.05 on the drying outcome. These findings are in accordance with Bauer et al. (2012). They also reported the positive correlation between a cultivation pH at the optimum and a resulting higher drying resistance for *Bifidobacterium lactis* due to a different structure and composition of the cell membrane. The composition of fatty acids in the phospholipid bilayer resulted in differently stable membranes during drying. To validate those results for the strain used in this study, the membrane preservation was measured. For *B. longum* Reuter 1963, free acidification led to a preservation of only 1.0% of membranes after drying, whereas cultivation at pH 6.0 induced more stable cell membranes, resulting in a membrane preservation of 43%. Survival rate and residual cell membrane integrity under different cultivation conditions here also correlate directly.

**Table 2:** Survival rates for the tested influencing factors during cultivation, drying, and storage; statistical analysis was done with a one-way ANOVA (*p* ≤ 0.05), followed by a Tukey post hoc test

Influencing factor		Tested parameter	Survival rate (%)
Cultivation	pH control^1^	pH 6.0 Free acidification	40 ± 15 0.5 ± 0.4
Drying	Shelf temperature (°C)^2^	+ 25 + 35 + 40	17 ± 2 40 ± 15 6 ± 0.7
	Type of protectant^3^	Without Glucitol Trehalose Maltodextrin	40 ± 15 34 ± 3 17 ± 7 43 ± 3
	Concentration of MD addition (% BDM)^3^	10 25 50 75 100	28 ± 3 26 ± 15 31 ± 9 49 ± 13 43 ± 3
Storage (70 days)3	Addition of protectant and ambient temperature (°C)^3^	4 °C without protectant 4 °C with 75% BDM MD 20 °C without protectant 20 °C with 75% BDM MD	87 ± 6 100 ± 15 28 ± 1 57 ± 7

^1^Drying process: 3700 Pa, 24 h, − 10/+ 10/+ 35 °C, no protectant

^2^Drying process: 3700 Pa, 24 h, − 10/+ 10/*adapted due to information in table*, no protectant

^3^Drying process: 3700 Pa, 24 h, − 10/+ 10/+ 35 °C

### Effect of the drying process

#### Influence of the shelf temperature during the third drying step on survival

The freeze-drying step followed cultivation, harvesting, and concentration. Cells cultivated at the optimal pH of 6.0 were used for all the following experiments since they showed a significant higher rate of survival. A constant chamber pressure of 3700 Pa was used for drying. The shelf temperature *T*_Shelf_ was held at − 10 °C for 12 h and then raised to + 10 °C for 6 h. During this period, the water was mainly removed due to sublimation. Following this drying period, the residual water content was 12.9% corresponding to an *a*_w_ value of 0.274. At this time, most of the surface water had been removed (Higl et al. 2008). To ensure storage stability, a third drying step, at a higher shelf temperature of + 25, + 35, or + 40 °C for another 6 h, followed to remove water within the cell and cell membrane. In total, the time allowed for each drying process was 24 h. The aim of this step was to determine a temperature that yielded a stable dry product. To prevent protein denaturation and loss of activity, the temperature must not exceed the physiologically optimal temperature of the strain of + 40 °C (Kiviharju et al. 2005). The lower limit of + 25 °C was chosen according to previous freeze-drying studies involving different starter cultures (Ambros et al. 2018b). During the drying process, the temperatures of the shelf and inside the product were controlled with internal sensors to avoid exceeding the set temperature limits. At the end of each drying, the temperature difference Δ*ϑ*, according to Eq. 1, was 0. Hence, the drying came to an end as no energy was brought into the product anymore by the shelf.

Heat and dehydration are obviously the main damaging factors for microbial cells when dried. Both can cause the permanent loss of the cells’ viability. Table 2 shows the survival rate at different temperatures of the final drying step: at + 35 °C, a maximum is reached.

At this temperature, a low residual moisture content of 5.5% and a higher survival rate of 40% compared with + 25 (6% SR) and + 40 °C (17% SR) were achieved at the end of the process. Here, an *a*_w_ value of 0.097 was reached, indicating a stable product. Measured by Δ*ϑ*, no more energy was added by heat at the end of any of the three drying setups. Nevertheless, the *a*_w_ value at + 25 °C of 0.198 indicates an unstable product. Microorganisms are most sensitive in an *a*_w_ region of 0.3 to 0.5, as there is not enough water anymore to keep them in a agile state, but still too much water to bring them into a preserved state (Higl et al. 2008). Here, the *a*_w_ value is lower, but somehow not low enough to keep the cells in a stable state. Consequently, at + 25 °C, the drying progress seems to be not efficient enough. This means that the cells were kept for longer times at higher or medium water content levels, where their sensitivity against processing stress is high. In total, the influence of a shelf temperature of + 35 °C on the survival rate is significant based on a *p* value of 0.05. It seems to be efficient to prevent major heat damage compared with + 40 °C. It is also high enough to minimize damage through insufficient drying compared with a shelf temperature of + 25 °C.

#### Influence of the addition of protectants on survival after freeze-drying

Another option to raise the cells’ survival rate during probiotic manufacture is to use protectants. Based on results obtained with lactobacilli and bifidobacteria, maltodextrin (MD), glucitol (S), and trehalose (T) were chosen as candidates for *B. longum* Reuter 1963 (Ambros et al. 2018a; Oldenhof et al. 2005; Sohail et al. 2013). Each protectant was added before drying at a concentration of 100% related to the bacterial biological dry matter (BDM; w/w) content.

**Fig. 3:**
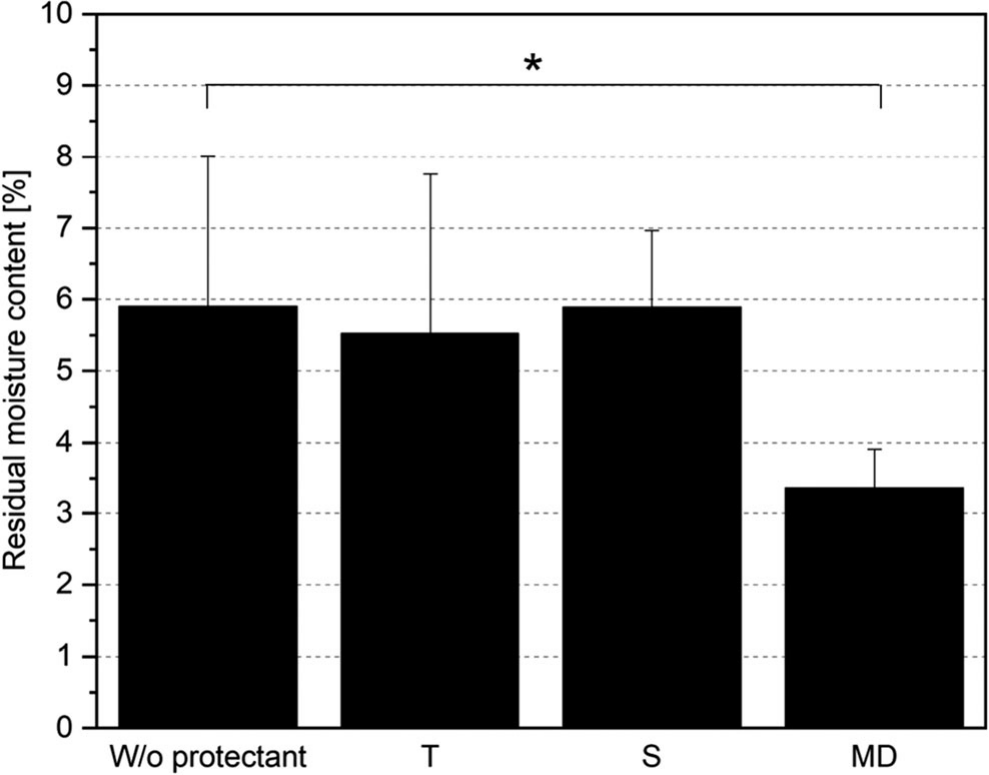
Residual moisture content for cell suspensions with an addition of 100% BDM maltodextrin, glucitol, or trehalose as well as without protectant; significant differences between data based on a one-way ANOVA (*p* ≤ 0.05), followed by a Tukey post hoc analysis are marked with an asterisk

The results in Table 2 indicate little, if any, protective effect of the added protectants in terms of cell survival. No significant statistical difference was detected. Adding maltodextrin (MD) resulted in a survival rate of 43%, comparable with survival without protectant (40%). Adding glucitol (34%) and trehalose (17%) resulted in reduced survival rates. Considering only survival rate, no positive protective effect could be demonstrated through the addition of protectants. However, the added protectants were found to significantly influence the residual moisture content after drying (Fig. 3). A moisture content of 3.4% was obtained for samples containing MD compared with samples without added protectant (5.5%), glucitol (5.9%), or trehalose (5.5%). Based on a one-way ANOVA followed by a Tukey post hoc test, there was a significant difference in the moisture content between samples without protectant and maltodextrin. Regarding the measured *a*_w_ values of 0.109 (no protectant), 0.101 (MD), 0.194 (S), and 0.224 (T), a similar trend can be observed. This may be since trehalose and glucitol are hygroscopic. As a result, the moisture will be higher so that inactivation is also consistently higher leading to the pronounced difference in the survival rate. MD may be able to protect through a transition of the dried suspension into the glassy state, which is important to achieve a high storability (Santivarangkna et al. 2008). Measurements with differential scanning calorimetry showed that the final product had a glass transition temperature of + 40 °C. Therefore, it remains in a glassy state after drying, resulting in higher survival rates due to the decreasing rate of chemical reactions. Consequently, glucitol and trehalose do not seem to be appropriate protectants for *B. longum* Reuter 1963 in a concentration of 100% BDM. Concerning survival rate, adding MD has no effect, but resulted in a lower residual moisture content.

To further study this effect, MD was tested as a protectant at concentrations of 10, 25, 50, 75, and 100% BDM to assess at what concentration this effect was occuring. Measured by Δ*ϑ*, the 24-h drying program was known to be sufficient for all concentrations. Table 2 shows that with increasing MD concentration, the survival rate rises from 28% (10% BDM MD) to 49% (75% BDM MD).

The residual moisture content decreased with increasing MD concentration: 7.3% (10% BDM MD), 5.6% (25% BDM MD), 4.8% (50% BDM MD) to 3.0% (75% BDM MD). Consequently, with an addition of MD, no significant difference in SR, but in moisture content, was detected. In this study, the addition of 75% BDM MD was found to be sufficient.

As shown here, MD prevents inactivation, resulting in a survival rate as high as without MD. Additionally, the moisture content is significantly lower, which should result in a higher SR during storage. These results appear surprising, as the survival rate normally decreases with a diminishing moisture content (Higl et al. 2008). The reason for the here obtained results may be the protective effect of MD. The MD used in this study has a dextrose equivalent of 4.0 to 7.0, which leads to a degree of polymerization around 22 and an average molecular mass of about 2900 to 3500 Da (Dokic et al. 2004; Rong et al. 2009). MD molecules with this physical properties lead to high viscosities in the surrounding of the cells (Dokic et al. 2004). This may support the transition of the product in the glassy state and should therefore produce a more stable cell membrane and a higher survival rate. Furthermore, a protection with an effect similar to the water replacement theory could be considered. Some researchers stated that the MD molecule is too big to interact with the cell membrane of lactobacilli (Santivarangkna et al. 2008). On the other hand, others showed the interaction with phosphatidylcholin membranes depending on the size of the MD molecule (Koster et al. 2003). A molecular mass of 1000–5000 Da and a degree of polymerization of 6–27 are the upper size limits for MD to be integrated in phosphatidylcholine membranes on the course of drying. In this study, MD may offer protection since bifidobacteria have a different composition of phospholipids in the cell membrane compared with lactobacilli. Lactobacilli membranes contain mainly phosphatidylglyceroles and lack nitrogen-containing phospholipids as phosphatidylcholin (Drucker et al. 1995; Exterkate et al. 1971; Novik et al. 2006), whereas bifidobacteria have a high content of phosphatidylcholin in their membranes (Novik et al. 2006). This may result in a different interaction of MD with the cell membrane compared with lactobacilli strains, explaining the divergent results in this study. Additionally, the transition into the glassy state of products dried with MD will also have a protective effect due to the decreased rate of chemical reactions. Trehalose is known to interact with the carbonyl and phosphate groups in the cell membrane and is able to replace water molecules from the lipid headgroup (Villarreal et al. 2004). Nevertheless, in this study, a protection was not observed. A possible reason for that effect could be the different composition of cell membranes of lactobacilli and bifidobacteria, as described above. Further, glucitol is not able to protect lactobacilli during drying, although the reason is not clear yet (Carvalho et al. 2003). For bifidobacteria, also no protective effect was observed. However, the exact interaction between cells, cell membranes of *B. longum* Reuter 1963, and protectants is not fully understood to date. It needs further investigation to confirm the exact molecular mechanisms behind its protective or non-protective effects.

### Correlation of membrane preservation and cell survival

Inactivation during probiotic production can occur in different ways. During the drying step, dehydration and denaturation of proteins have the largest influence on cell survival. Dehydration leads to a exponential decrease of survival rate and membrane damage (Higl et al. 2008). As shown previously, the cell membrane is the component that is subject to the most damage during preservation. To verify this effect for the data obtained in this study, the membrane integrity of the dried cells was measured by flow cytometry. Table 3 shows the correlation of survival rate and membrane preservation for all drying setups. With a *R*^2^ of 0.85, membrane preservation had a exponential impact on the survival of cells. Taking into consideration that the main part of damage occurs during drying supports the membrane integrity hypothesis. Accordingly, an intact cell membrane, shown by a high membrane preservation, after drying will result in a high survival rate. Additionally, further effects like denaturation may also have a smaller impact.

**Table 3:** Fitted correlation of survival rate over membrane preservation; fitting was done with OriginPro 2019 according to the shown statistical parameters

Process step	Correlation	*R*^2^
Drying	Exponential: *y* = *y*_0_ + *A*_1_ · ${e}^{\frac{x}{t_1}}$ *y*_0_ = 0.26 ± 1.19 *A*_1_ = 0.05 ± 0.05 *t*_1_ = 7.40 ± 1.30	0.85
Storage	Linear: *y* = *mx* *m* = 1.07 ± 0.03	0.97

### Effect of storage on cell survival

Cultures are dried to increase their storage stability. In this study, a short-term storage of 70 days was investigated to reveal storage-related effects on inactivation. The impact and correlation of storage temperature and addition of maltodextrin were investigated. In parallel, the influence of membrane integrity on the survival rate was assessed to allow an explanation for inactivation. The dried samples were stored for 70 days at + 4 °C (refrigerated conditions) and + 20 °C (ambient conditions), with and without an addition of 75% BDM MD. Thus, four different conditions were evaluated.

To analyze only the influence of storage, cell count, and residual moisture content, refer to the values after drying. Since the storage bags were flooded with nitrogen prior to sealing, there was no humidity or oxygen inside the containers. Hence, the moisture content was constant over the 70-day process (data not shown). After storage, the moisture content was between 3.3% (+ 20 °C, with MD) and 6.5% (+ 4 °C, without MD), which is similar to the values after drying. The storage temperature itself has no influence on the moisture content as it stays constant over the whole process. Regarding survival rate, another trend was observed, as shown in Table 2. After 70 days of storage, the survival rate at + 4 °C was 87% (without MD) and 100% (with MD) related to the cell count after drying. At + 20 °C, the survival rate was significantly lower with values of 28% (without MD) and 57% (with MD). For *B. longum* Reuter 1963, the survival rate is higher at lower temperatures. These findings are in consistence with the studies of Abe et al. (2009).

Comparing the addition of MD within one storage temperature, no significant influence was detected (*p* > 0.05). Thus, as Table 2 shows, the storage temperature was even more critical than the addition of MD and had a significant influence based on a *p* value of 0.05. Additionally, the effect of MD is more pronounced with increasing temperature. At + 4 °C, the SR can be increased by 13%, whereas at + 20 °C the SR is increased by 29% when adding the protectant. The higher SR caused by an addition of MD is probably due to the lower moisture content over storage time. No cells were lost over 70 days of storage when the freeze-dried suspensions were stored at + 4 °C with added MD. Furthermore, MD was able to compensate the effects of non-ideal conditions, such as a higher storage temperature of + 20 °C compared with + 4 °C.

**Fig. 4:**
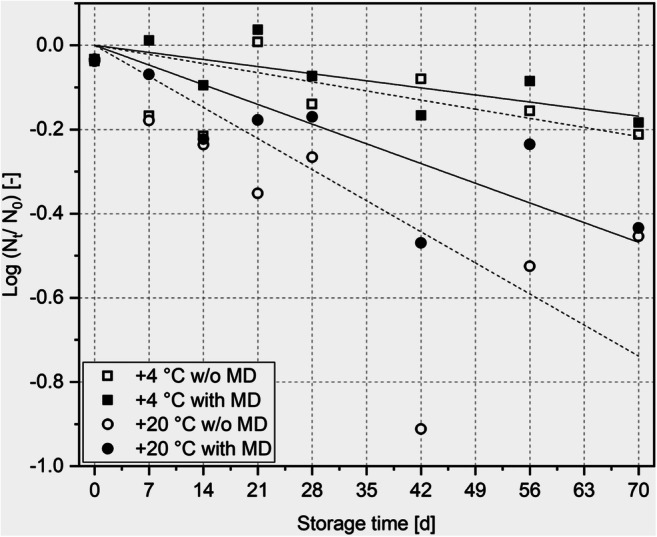
Relative viabilities of *B. longum* Reuter 1963 after freeze-drying as a function of storage time; standard deviations are not plotted for a clearer graph, but taken into consideration for calculation

To monitor inactivation of *B. longum* Reuter 1963 over the whole storage process, the inactivation rate constant was calculated according to Eq. 4 The logarithmic progression of cell count over storage time is shown in Fig. 4. In general, a low inactivation rate constant predicts fewer inactivated cells during storage.

**Fig. 5:**
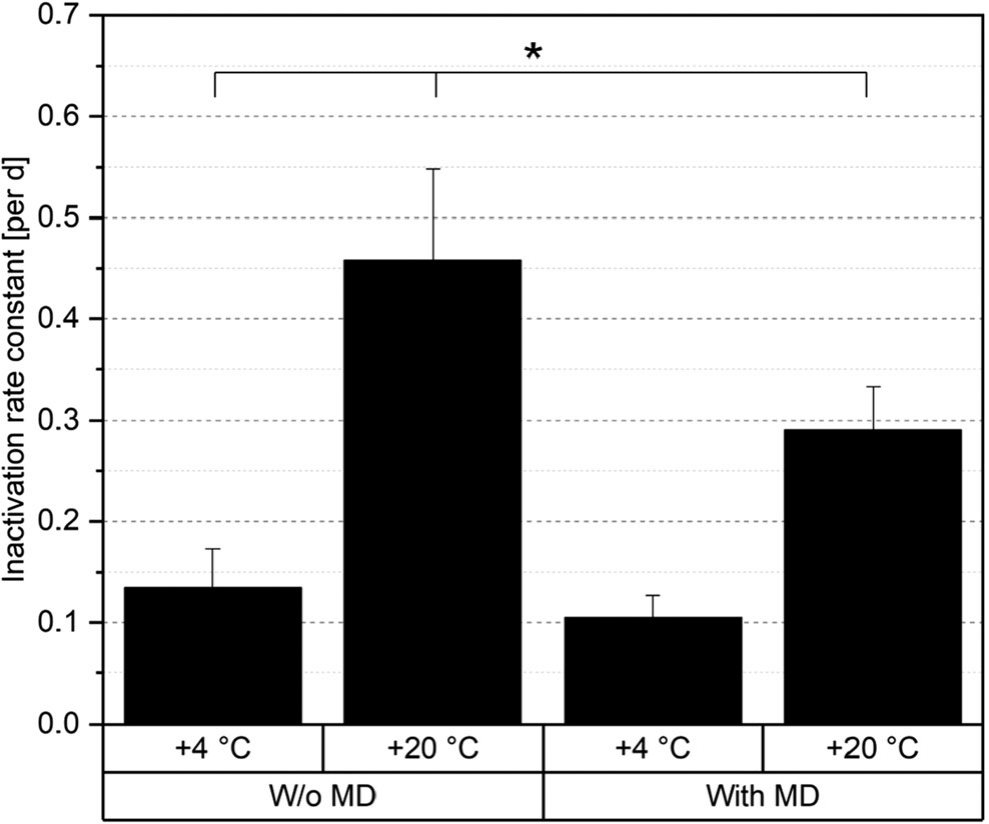
Inactivation rate constants of the four storage conditions, significant differences between data based on a one-way ANOVA (*p* ≤ 0.05), followed by a Tukey post hoc analysis are marked with an asterisk

It was visible that the inactivation rate constant was higher when samples were stored at + 20 °C (Fig. 5). The influence of an addition of MD (0.29 per days) or absence of MD (0.46 per day) at + 20 °C was far smaller. Here, the influence of the higher storage temperature caused a higher damage than the lack of MD. The lowest level of inactivation was achieved at + 4 °C with an addition of 75% BDM MD. In this case, the constant was 0.10 per day. Hence, no significant difference could be seen for the inactivation rate constant at + 4 °C, but at + 20 °C.

Also during storage, the influence of membrane integrity on cell survival was investigated. For each point of time during storage, survival rate and membrane preservation were determined. Here, the survival rate is linearly affected by the membrane preservation (*R*^2^ = 0.97). Consequently, cell loss during storage is probably due to leakage in the cell membranes. Storing cells with a high membrane integrity results in a high survival rate during the storage process.

Compared with results reported in literature, the data obtained in this study show a lower inactivation level. Shamekhi et al. (2013) stored freeze-dried *B. longum* Reuter 1963 cells without protectant at + 4 °C and + 25 °C. In their study, the cell count decreased between 1 (+ 4 °C) and 3 (+ 25 °C) log units after 70 days. The higher survival of the cells in the present study may result from storage in air-tight aluminum pouches and therefore the absence of oxygen and a low water activity in the environmental air. A low water activity in the environmental air in the pouches during storage is linked to a low inactivation rate and, consequently, a high survival rate (Higl et al. 2008).

### Development of cell count over the whole production process

The aim of this study was to observe and to increase *B. longum* Reuter 1963 survival rates over the whole production process, including cultivation, cell harvest, drying, and storage. By regarding the whole process, we are able to detect harmful process steps and determine how cell count evolves over the whole process. It is recommended to apply probiotics in a concentration of 10^10^ CFU/mL to see a permanent abundance of the strain in the microbiome and have a positive effect on consumers’ health (Knorr 1998; Saxelin 1997). Hence, we set out this number as desired cell count.

**Fig. 6:**
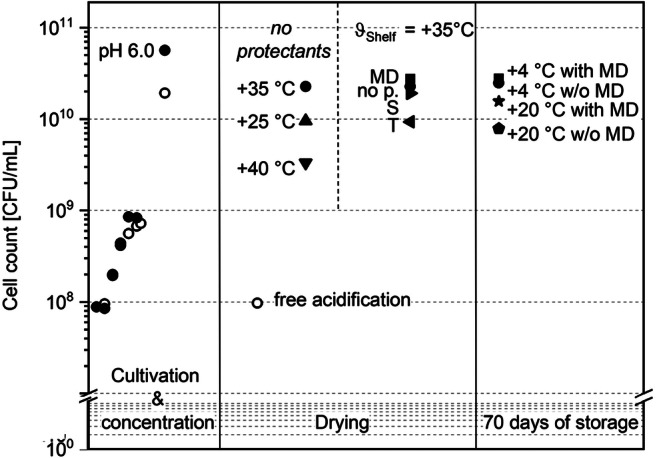
Cell count over the whole process: inoculation and cultivation, concentration, after drying and 70 days of storage; full symbols refer to experiments that were carried out with cells cultivated at pH 6.0, whereas hollow circles stand for free acidification

Figure 6 shows the cell count across the whole process, from cultivation to drying and storage, with all tested influencing factors. All cultivations got started with an initial cell count of 6 · 10^7^ CFU/mL. Figure 6 shows the increase of cell count during cultivation to 8 · 10^8^ CFU/mL (pH 6) and 7 · 10^8^ CFU/mL (free acidification), followed by concentration, which leads to a final cell count of 2 · 10^10^ CFU/mL (free acidification) and 6 · 10^10^ CFU/mL (pH 6.0). As described above, when cultivating without pH regulation, the survival rate was only 0.5% (1 · 10^8^ CFU/mL) after drying, whereas 40% of the cells cultivated at pH 6 survived with a cell count of 2.4 · 10^10^ CFU/mL, respectively. As the survival rate for cells cultivated at free acidification dropped by more than 2 log units after drying and less than 10^10^ CFU/mL survived, there are too less cells for application. Consequently, no further experiments were performed for drying and storage. For drying and storage, all experiments were performed with cells cultivated at pH 6.0. During drying, the shelf temperatures were varied between + 25, + 35, and + 40 °C. For these experiments, no protectants were added. Afterall, a cell count of 10^10^ CFU/mL, required for a stable abundance in the microbiome, can only be reached when a shelf temperature of + 35 °C is applied. Higher or lower temperatures result in a lower cell count. In comparison with that, another set of drying experiments was conducted at *ϑ*_Shelf_ = + 35 °C, while different protectants were added. As explained above, a shelf temperature of + 35 °C and an addition of 75% ΒDM MD lead to the highest survival (49%) with a cell count after drying of 3 · 10^10^ CFU/mL. These settings were used for the storage experiments. During 70 days of storage, only a small decrease in cell count was observed. During storage at + 20 °C without MD, less than 10^10^ CFU/mL survived. On the other hand, no drop in cell count was observed for cells stored at + 4 °C with an addition of MD. Consequently, a selection of appropriate storage conditions together with an adapted and improved drying process can prevent complete losses in cell count. Given that the cell count after concentration was referred to as 100%, after drying, between 50% (pH 6, + 35 °C shelf temperature, 75% BDM MD) and 94% (pH 6.0, + 40 °C shelf temperature, no protectant) of cells were lost. At the end of the process, after drying and storage, total cell loss was between 50% (+ 4 °C, with MD) and 86% (+ 20 °C, without MD). The most appropriate conditions were found to include cultivation at pH 6.0, a shelf temperature of + 35 °C, the addition of 75% BDM MD, and storage at + 4 °C. Under these conditions, the entire inactivation through drying was 50% (50% SR, respectively) and 0% through storage. Consequently, drying leads to an inactivation of a higher number of cells than storage.

## Conclusions

In the current study, different factors influencing *B. longum* Reuter 1963’s survival were assessed. From the results obtained, we can conclude that the here investigated steps (cultivation, drying, and storage) strongly influence the process. The choice of cultivation conditions here provided the biggest potential of increasing the survival after drying. The choice of appropriate cultivation conditions is important as cell membrane stability during drying correlates directly with drying resistance and survival rate. Supplementing other studies, where the effects of cultivation, drying, and storage were assessed separately, we here show how the factors involved in inactivation influence the particular process step and how some factors interact during storage. Furthermore, with choosing appropriate settings during drying, an inactivation of cells during storage can be prevented completely in the here investigated period of time. Membrane integrity has a high impact on survival during both drying and storage, as cells with leakage in the membrane fail to survive the process. Regarding the practical implication of the here shown results, cultivation at pH 6.0, a drying process of 3700 Pa, 24 h, at − 10/+ 10/+ 35 °C shelf temperature with an addition of 75% BDM maltodextrin, and a storage at + 4 °C result in the here investigated highest survival of cells. In future studies, these conclusions should be confirmed involving investigations of further probiotic strains from the human intestinal microbiome. Additionally, the interaction of maltodextrin with cell membranes needs to be the interest of following works to assume and verify the here obtained results.
